# The Beneficial Effect of Traditional Chinese Exercises on the Management of Obesity

**DOI:** 10.1155/2020/2321679

**Published:** 2020-10-01

**Authors:** Yuan Qin, Weiyi Xia, Wei Huang, Jing Zhang, Yi Zhao, Min Fang

**Affiliations:** ^1^Shanghai University of Traditional Chinese Medicine, Shanghai, China; ^2^Shuguang Hospital Affiliated to Shanghai University of Traditional Chinese Medicine, Shanghai, China; ^3^Shanghai Green Valley Pharmaceutical Co., Ltd., Green Valley Research Institute, Shanghai, China

## Abstract

This paper systematically reviewed the clinical update of traditional Chinese exercises in the treatment of simple obesity in recent years and discussed their specific advantages in this aspect. This review focused on several typical traditional Chinese exercises, namely, Tai Chi, Ba Duan Jin, Yi Jin Jing, Wu Qin Xi, Shaolin Neigong, and Liu Zi Jue, which all showed clinical beneficial effect on the treatment of simple obesity with their own characteristics. To optimize the clinical therapeutic effect of these traditional Chinese exercises, we need to seek the most appropriate exercise or the combo exercise based on the characteristics of different obese population, to improve the efficiency of weight loss, reduce sports injury, and consolidate the therapeutic effect. In the future, we need to further evaluate the efficacy of sitting exercise, lying exercise, and static training in the treatment of simple obesity, subdivide the treatment population, and explore the working mechanism of these traditional Chinese exercises.

## 1. Introduction

Obesity is a clinical syndrome in which the body fat content is too high and/or the distribution is abnormal under the action of genetic and environmental factors, so that the actual body weight exceeds 20% of the ideal body weight and can be complicated with cardiovascular disease and a variety of endocrine and metabolic disorders [1].

Data from the Blue Book on Obesity Prevention and Control in China in 2019 show that the proportion of overweight and obesity in China is more than 40%, and 10 to 20% of children are obese or overweight [2]. Obesity not only affects the external image but also causes psychological problems such as depression [3]. Obesity in adolescents can cause chronic brain hypoxia and affect learning. Obesity is also a risk factor for chronic diseases such as cardiovascular disease, diabetes, and some tumors [4].

According to the management guidelines of the American Obesity Society, the treatments for obesity include dietary intervention, lifestyle intervention, drug treatment, and surgery [5]. Due to some nonideal situation including poor patient compliance, many adverse reactions, and easy rebound, there is an urgent need for a healthier, simpler, and more sustainable treatment.

Exercise is a common way to lose weight, and scholars at home and abroad are exploring and establishing scientific and effective exercise way [6].

Traditional Chinese exercises are an ancient way of exercise and fitness, which have been reported to have unique advantages in the treatment of obesity. In this paper, we summarize the related research on the traditional Chinese exercises in the treatment of obesity in recent years to provide reference for clinical application and experimental research.

## 2. Tai Chi

Tai Chi is a form of sport that includes oriental culture, with the characteristics of both internal and external practice, firmness and softness, and slow and light spirit, which is the first batch of national intangible cultural heritage in our country [7]. Tai Chi is a medium- and low-intensity aerobic project [8]. A large number of studies have shown that Tai Chi has a significant effect in the treatment of the onset and progression of obesity.

Studies have found that Tai Chi exercise can gradually restore the normal activity of abnormally expressed AMPK genes in obese patients [9] and significantly reduce plasma neuropeptide Y [10], especially in patients with high triglycerides. Tai Chi can also improve the level of serum ghrelin in high-risk groups of metabolic syndrome [11], regulate the feeding center, reduce body weight, reduce waist circumference, improve blood lipid indexes, and eliminate the risk factors of metabolic syndrome.

The rapid proliferation of adipocytes caused by the proliferation and differentiation of preadipocytes is one of the possible mechanisms of obesity [12]. It has been found that the growth of hormone GH and catecholamine can inhibit the differentiation of preadipocytes in different degrees [13, 14]. Long-term Tai Chi exercise can significantly increase the GH content of growth hormone [15], reduce the concentration of catecholamine, and increase the level of catechol statin [16]. In the study of lipid metabolism and related hormones in Tai Chi, it was found that after Tai Chi exercise, the indexes of total cholesterol (TC), triglyceride (TG), and low-density lipoprotein cholesterol (LDL C) decreased, while high-density lipoprotein cholesterol (HDL C) increased [17]. Tai Chi can significantly reduce the levels of serum insulin and leptin [18] and increase the level of adiponectin, a special active peptide secreted by adipocytes [19]. It can enhance insulin sensitivity and promote the oxidation of fatty acid in the body. It can inhibit inflammation to a certain extent [20]. Tai Chi exercise can reduce fasting blood glucose, 2-hour postprandial blood glucose, and glycosylated hemoglobin, stabilize human blood glucose level [21], and has a good balance effect on human energy metabolism.

A study found that after 6 months of Tai Chi exercise [22], the number of intestinal *acidophilus*, *Lactobacillus*, and *Bifidobacterium* increased significantly in obese elderly people and positively correlated with the training time. Through the effect on intestinal flora, the transformation and utilization of cholesterol was strengthened, and the level of blood lipids in the human body was ameliorated. In addition, Tai Chi exercise can also reduce lipid peroxidation, significantly reduce hs-CRP, IL-6, TNF-*α*, and other inflammatory indicators, and ameliorate the microinflammatory status caused by obesity [23].

## 3. Ba Duan Jin

Ba Duan Jin has the advantages of softening the tendons and strengthening the bones, nourishing the qi and enhancing strength, promoting qi and invigorating blood circulation, and coordinating the functions of all the organs. After the reorganization by the State General Administration of Sports, the current version has a total of eight movements, each with a different focus, which can be practiced according to a certain viscera or disease syndrome [24].

Studies have shown that Ba Duan Jin exercise has a good effect on weight loss and good posture by losing weight and reducing waist and hip circumference [25]. Ba Duan Jin exercise could effectively reduce fasting blood glucose, serum insulin concentration, and insulin resistance index [26] and reduce the concentration of total cholesterol, triglycerides, and free fatty acid, and the level of leptin decreased with the decrease of blood lipid level and obesity index (body weight, BMI, and percentage of body fat), while adiponectin level was negatively correlated [27]. There is evidence showing that after practicing Ba Duan Jin, the number of *Bifidobacterium*, *Bacteroides*, and *Lactobacillus* increased significantly, while *Enterobacter*, *Clostridium*, and *Enterococcus* decreased significantly [28]. By regulating the structure of intestinal flora in the elderly, fasting-induced fat factor (fasting-induced adipocyte factor, FIAF) [29] can be affected and triglyceride deposition and storage can be reduced. The inflammatory markers (CRP, IL-6, and TNF-*α*) have been found decreased significantly in Ba Duan Jin participants [30]. However, its inhibitory effect on obesity still needs to be verified by further experiments.

## 4. Yi Jin Jing

Yi Jin Jing is a fitness exercise characterized by improving the function of muscles and bones, stimulating the circulation of the blood, and causing the muscles and joints to relax. After the reorganization by the State General Administration of Sports, the current version has a total of 12 postures, with the combination of movement and stillness. The movement requires stretching tendons and bones, fully flexion and extension, abduction, adduction, torsion of the body, etc., in order to pull the muscles and fascia of various parts of the human body, as well as the tendons, ligaments, joint capsules, and other connective tissues, which can play a positive role in body shape and physiological function. Persistent exercise can strengthen the muscles and bones and benefit the internal viscera [31].

Practicing Yi Jin Jing can also regulate glucose and lipid metabolism. The study found that 6 months of Yi Jin Jing exercise can significantly reduce the levels of serum TC, TG, and LDL C and increase the level of HDL C [32]. Long-term persistence can reduce the levels of fasting blood glucose and insulin and reduce insulin resistance accompanied by the decrease of inflammatory reactions [33]. The percentage of CD4+ T cells, CD8+ T cells, and NK cells and the levels of IL-2, IL-6, and IFN-*γ* in the immune indexes of the elderly were significantly increased after 24 weeks of practicing Yi Jin Jing [34], so as to improve the immunity of the elderly.

## 5. Wu Qin Xi (Five Fowl Opera)

Wu Qin Xi is a set of traditional aerobics exercise created by Hua Tuo, a famous physician of the Eastern Han Dynasty, who combined the posture and movements of the tiger, deer, bear, bird, and ape, with the theory of the five elements of traditional Chinese medicine. It is the earliest guiding technique recorded so far and is the world-class intangible cultural heritage [35].

Wu Qin Xi exercise could reduce the serum levels of TC, TG, and LDL C and increase the level of HDL C in elderly women and significantly improve the level of blood lipids after 6 months of practice [36]. After 24 weeks of practice of Wu Qin Xi, blood sugar and glycosylated hemoglobin levels decreased by 7.14% and 13.23%, respectively, which effectively controlled blood sugar levels [37]. At the same time, Wu Qin Xi practice can also reduce the levels of leptin and insulin and increase the level of adiponectin [38, 39]. After practicing Wu Qin Xi for 2 months [40], the amount of *Bifidobacterium*, *Lactobacillus*, *Bacteroides*, *Clostridium*, SCFAs, GLP-1, and GLP-2 in the intestine of the practitioners increased, and this effect increased with time. Wu Qin Xi reconditioned human microecology, improved the structure of gut microbiota, reduced intestinal permeability, and regulated human metabolism. Wu Qin Xi practice improved the immune function of the middle aged and elderly [41, 42]. It was found that the activity of NK cells and the ratio of CD+4/CD+8 increased significantly after exercise, which played a positive role in regulating immune balance.

## 6. Shaolin Neigong

Shaolin Neigong is an important part of internal Qigong massage therapy, that is, Shanghai intangible cultural heritage. This exercise pays attention to strength and endurance with the lower limb standing crotch posture as the basic exercise combining with the upper limb movement. It is characterized by skeletal muscle static contraction exercise [43], emphasizing the “static resistance” of the lower limbs and the “internal strength” of the upper limbs [44].

The effective rate of Shaolin Neigong in the treatment of simple obesity among college students is 85.7%, which can significantly reduce the weight, BMI, and waistline and hip circumference of practitioners [45]. Shaolin Neigong can reduce the insulin resistance index and improve the sensitivity of the body to insulin [46]. The exercise frequency test of Shaolin Neigong showed that when the exercise frequency was 1–4 times a day, the subjectsʼ levels of FPG, 2hPBG, and HbA1c were improved [47].

It was found that the inverted suspension of rats could simulate the static characteristics of Shaolin Neigong [48]. It was found that static training could increase the transcription level of the hypothalamic *POMC* gene and the content of *β*-endorphin in the hypothalamus [49], thus reducing the appetite for food. A study reported that static training combined with massage therapy could increase the protein content of skeletal muscle, reduce the excretion of creatinine (CRE) and 3-methylhistidine (3-MH), and regulate protein metabolism [50]. Qigong training can partially eliminate the RNA interference of the PGC-1*α* signal pathway [51], suggesting that this kind of static training can facilitate the activation of the PGC-1*α* signal pathway, induce the expression of PGC-1*α*, and consequently regulate central appetite.

## 7. Liu Zi Jue

Liu Zi Jue refers to a breathing practice of exhale and inhale in six different ways, namely, si, xu, xi, he, hu, and chui, in order to mobilize the function of various organs to activate qi and blood circulation. It is an ancient health-preserving method, which can make the breath natural, relax the body, and calm the mind.

Comparing the effects of different traditional exercises on fasting blood glucose, it showed that after 3 months of exercise, Wu Qin Xi, Ba Duan Jin, and Liu Zi Jue all could effectively reduce fasting blood glucose (FPG), and among them Liu Zi Jue showed the strongest effect [52]. After practicing Liu Zi Jue [53], both body weight and BMI showed a trend of decrease, and the grip strength, bouncing strength, fast walking, flexibility, and other exercise abilities of elderly were all improved in varying degrees.

## 8. Others

Comparing the effects of Tai Chi, Baduanjin, Yi Jin Jing, and Wuqinxi on the immune function of the elderly showed that they all can significantly increase the immune indexes, including CD3+, CD4+, CD8+, and NK cells. Among them, Tai Chi was the best in improving CD4+ and NK cells, and Ba Duan Jin was the best in improving CD3+ and CD8+ cells [54]. Both “Mawangdui Dao Yin” and fitness Qigong “Da Wu” can reduce TG, TC, and LDL C and increase HDL C to some extent [55, 56]. Some scholars combined Mawangdui Dao Yin, Tai Chi, Shi Er Duan Jin, Dao Yin health-preserving gong, and Da Wu to sort out a new set of Qigong exercise [57] and found that this exercise also can regulate lipid metabolism. In the study of the Pigu weight loss experiment, it showed a significant effect on weight loss [58].

Other traditional Chinese exercises that have not been verified by experiments, such as “weight loss and antiaging Qi gong” [59], Huichun Gong [60], Tiaoxi Zhuji Gong [61], Xiaoyao walking, Guanyin Gong, stepping abdomen beating Gong, abdominal retraction Gong, palm closing Gong [62], simple weight loss Gong, relaxation weight loss Gong, Xiao Zhou Tian loss Gong, Yannian Jiuzhuan Gong, Jade toad Xi Zhen Gong, Guanyin lotus seat, Jade toad wave turning Gong, standing pile, Longmen bodybuilding, and longevity Gong [63], are clearly stated that they have a certain degree of effect on weight loss. It is worth further exploring.

## 9. Summary

At present, there are many studies on the traditional Chinese exercises in the treatment of obesity, which reveal good results from different angles. Among these exercises, it seems that Tai Chi is the most widely studied one. In terms of the research direction, they are mostly focused on the effect of Qigong exercises on the level of glucose and lipid metabolism, but less on central feeding regulation, insulin, leptin, and adiponectin. In recent years, gut microbiota and immune functional regulation have attracted more attention. Compared with modern medicine, traditional Chinese medicine pays attention to personalized therapy based on the difference of patients and their different syndromes. In terms of obesity, it has the characteristics of wide age span, different degrees of obesity, and always has one or more complications. The traditional Chinese exercises mentioned in this paper not only have the commonness of weight loss and fat reduction, but also have their own characteristics, which can be selectively used for different types of obese patients.

Slow movement with breathing is what the above traditional methods have in common. When practicing Tai Chi, Ba Duan Jin, and Wu Qin Xi, the big movements of the upper and lower limbs cooperate with breathing. Tai Chi is beneficial to the overall balance, and Ba Duan Jin can increase lower limb strength and muscle content [64] and reduce fat content [65]. Wu Qin Xi can improve flexibility. The improvement of the heart rate of Tai Chi is better than that of Wu Qin Xi [66], while the exercise intensity is lower than that of Ba Duan Jin and Wu Qin Xi. For example, Tai Chi is suitable for patients with mild to moderate obesity, especially for the middle aged and elderly because Tai Chi belongs to low-intensity exercise and has advantages in controlling blood pressure, increasing bone mineral density, and reducing blood sugar, but it is not suitable for patients with severe obesity or knee osteoarthritis. Ba Duan Jin is more suitable for obese patients with endocrine and digestive system diseases such as cervical spondylosis and perimenopausal syndrome. Wu Qin Xi has certain advantages in improving the hyperlipidemia of adult obese patients.

The standing pile and movement of Yi Jin Jing Sutra pay attention to the stretching of muscles, standing pile after completing the action, and stretching when inhaling and relaxing when exhaling, which belong to intermittent-, medium-, and low-intensity training that can increase the muscle strength of the lower limbs of the elderly [67]. The standing pile and action of Shaolin Neigong emphasize the static contraction of muscles. Both the standing pile and the big movement should include contracting the muscles hard, breathing naturally, completing the action after standing pile, relaxing the whole body, and then continuing to the next standing pile or action after a short rest, which belongs to intermittent-, medium-, and high-intensity training. Shaolin Neigong reduces appetite and increases skeletal muscle content. Yi Jin Jing can slow down the physical decline and improve the symptoms of constipation. Shaolin Neigong has the characteristics of intermittent-, medium-, and high-intensity exercise, which is especially suitable for obese patients who are not easy to exercise because of excessive weight and also fit for the obese patients with poor cardiopulmonary function.

Liu Zi Jue can improve human body function through breathing exercises, slow down heart rate, increase vital capacity, stimulate vegetative nervous system, promote venous reflux, change hemodynamics, reduce hemorheological indexes, and improve the motility of visceral organs [68]. Liu Zi Jue is most suitable for obese patients with chronic obstructive pulmonary disease.

In addition, the sitting and lying exercises in the traditional method have also their own features, which are especially suitable for patients who cannot practice standing up; however, it still needs further study.

The traditional Chinese exercises have a long history, which contain the Chinese traditional fitness culture and also a concrete practice of the concept of “preventive treatment of disease” in traditional Chinese medicine. These exercises not only reduce fat and weight and adjust posture but also cultivate physical and mental health, which is beneficial. It is a simple and effective way to lose weight with low cost and is environment friendly.

## Data Availability

This article is a review article and does not contain relevant data.
